# Biofeedback combined with percutaneous electrical pudendal nerve stimulation for the treatment of low anterior rectal resection syndrome: a study protocol for a randomized controlled trial

**DOI:** 10.1186/s13063-024-08300-9

**Published:** 2024-07-02

**Authors:** Gaoyang Cao, Xinjie Zhang, Fei Wang, Da Man, Lijie Wu, Xuchu Pan, Shan Chen

**Affiliations:** 1 Department of Colorectal surgery, Sir Run Run Shaw Hospital, School of Medicine, Zhejiang University, Hangzhou, 310000 Zhejiang China; 2 Acupuncture and Moxibustion, The First Affiliated Hospital of Zhejiang Chinese Medical University Zhejiang Provincial Hospital of Chinese Medicine, Hangzhou, 310000 Zhejiang China

**Keywords:** Rectal cancer, Low anterior rectal resection syndrome (LARS), Percutaneous electrical pudendal nerve stimulation, Biofeedback, LARS score, Quality of life, Randomized controlled trial

## Abstract

**Background:**

Low anterior resection syndrome (LARS) is a distressing condition that affects approximately 25–80% of patients following surgery for rectal cancer. LARS is characterized by debilitating bowel dysfunction symptoms, including fecal incontinence, urgent bowel movements, and increased frequency of bowel movements. Although biofeedback therapy has demonstrated effectiveness in improving postoperative rectal control, the research results have not fulfilled expectations. Recent research has highlighted that stimulating the pudendal perineal nerves has a superior impact on enhancing pelvic floor muscle function than biofeedback alone. Hence, this study aims to evaluate the efficacy of a combined approach integrating biofeedback with percutaneous electrical pudendal nerve stimulation (B-PEPNS) in patients with LARS through a randomized controlled trial (RCT).

**Methods and analysis:**

In this two-armed multicenter RCT, 242 participants with LARS after rectal surgery will be randomly assigned to undergo B-PEPNS (intervention group) or biofeedback (control group). Over 4 weeks, each participant will undergo 20 treatment sessions. The primary outcome will be the LARS score. The secondary outcomes will be anorectal manometry and pelvic floor muscle electromyography findings and the European Organization for the Research and Treatment of Cancer Quality of Life Questionnaire-Colorectal 29 (EORTC QLQ-CR29) scores. Data will be collected at baseline, post-intervention (1 month), and follow-up (6 months).

**Discussion:**

We anticipate that this study will contribute further evidence regarding the efficacy of B-PEPNS in alleviating LARS symptoms and enhancing the quality of life for patients following rectal cancer surgery.

**Trial registration:**

Chinese Clincal Trials Register ChiCTR2300078101. Registered 28 November 2023.

## Introduction

### Background and rationale {6a}

Colorectal cancer (CRC) ranks as the world’s third most prevalent cancer, with the rectum affected in approximately half of the cases [1]. Surgical procedures remain the primary treatment for rectal cancer, offering opportunities for anus-preserving surgeries and improved quality of life using advanced medical technologies such as the Da Vinci robot [2]. However, postoperative complications include low anterior resection syndrome (LARS) that occurs in 25–80% of patients, characterized by fecal incontinence, urgency in bowel movements, and increased frequency of bowel movements [3]. Although these symptoms stabilize within 6 months to 1 year postoperatively, they often tend to persist [4, 5] and may significantly affect the patient’s life, sometimes even necessitating colostomy. Consequently, effective LARS treatments are urgently required.

LARS pathogenesis involves multiple theories, including anal internal sphincter dysfunction, reduced rectal sensitivity, the absence of the rectoanal inhibitory reflex, and reduced rectal compliance [6]. Nerve damage during the low anterior resection process might lead to new rectal innervation, exacerbating the LARS symptoms [7]. Current treatments encompass non-surgical options, such as pharmacotherapy, biofeedback, transanal electrical stimulation, and enema therapy, as well as surgical approaches, such as artificial sphincter replacement, colostomy, and neurostimulation therapy. Oral serotonin receptor antagonists, effective in treating diarrhea-predominant irritable bowel syndrome, are employed to alleviate LARS symptoms such as fecal incontinence and urgency;[8] however, further clinical trials are warranted for validation. Enema therapy effectively manages fecal incontinence but may have potential side effects, including rectal bleeding, perianal pain, anal fissures, abdominal discomfort, and colonic perforation [9]. Although artificial sphincter replacement is suitable for severe anal incontinence, the high cost and postoperative complications limit its application. Colostomy is the last treatment option in cases where other treatments fail; however, it imposes significant psychological stress on patients. Biofeedback may be effective in managing fecal incontinence by using transanal probes to track activity changes in the pelvic floor muscles, guiding patients through Kegel exercises, and delivering precise electrical stimulation for pelvic muscle recovery [10]. However, patients might find it difficult to follow Kegel exercise instructions, which could impede the treatment efficacy. Additionally, surface electromyography (EMG) stimulation via the anus provides an indirect and comparatively weaker therapeutic effect than deeper direct muscle stimulation, potentially offering less favorable outcomes [11].Recently, neural electrical stimulation therapies, such as sacral nerve stimulation and pudendal nerve stimulation, have become focal points in LARS treatment. Multiple clinical studies have affirmed their positive effects in treating fecal incontinence, LARS, and other defecation disorders [12, 13]. However, the implantation of electrodes in the body required in both stimulation methods poses issues, such as battery replacement, high costs, postoperative infections, and surgical failures [14]. Percutaneous electrical pudendal nerve stimulation (PEPNS) is an innovative technique developed by Wang Siyou [15]. It involves the insertion of extended needles through the lower sacral points on the skin confirmed on transverse computed tomography of the coccygeal apex, showing the needle tips located in the ischiorectal fossa proximal to the pudendal nerve within the Alcock canal [16]. The rectum and sphincter are regulated by nerves originating from the pelvic plexus located in the lower abdomen. This plexus includes both sympathetic fibers from the presacral nerves and parasympathetic fibers from the S2–S4 spinal segments, forming the neural basis for controlling functions of the rectum and sphincter muscles. The pudendal nerve is essential for the perineal sensation and voluntary movements of the pelvic floor muscles, especially the control of the external anal sphincter. It originates from the S2–S4 sacral spinal segments and divides into the following three branches in the Alcock canal: the inferior rectal branch, the perineal branch, and the dorsal nerve of the clitoris or penis. The inferior rectal branch innervates the external anal sphincter, levator ani, lower anal canal, and perianal skin [17]. The suggested mechanism involves continual electrical stimulation of the pudendal nerve alongside the merging of incoming nerve fibers from the anorectal region at the common neurons in the S2-S4 spinal segments, which regulate the visceral nerves [18, 19]. Moreover, pudendal nerve electrical stimulation can increase resting pressure in the sphincter, thus enhancing bowel control. [20]

Therefore, the application of PEPNS along with biofeedback could be an effective and economical non-surgical treatment for LARS. This study aims to conduct a randomized controlled trial (RCT) to compare the efficacy and safety of this combined approach with biofeedback alone.

### Objectives {7}

The aim of this study is to evaluate the short-term (1 month) and long-term (6 months) effects of B-PEPNS on alleviating symptoms of LARS and improving the quality of life for patients undergoing rectal cancer surgery.

### Trial design {8}

**Fig. 1:**
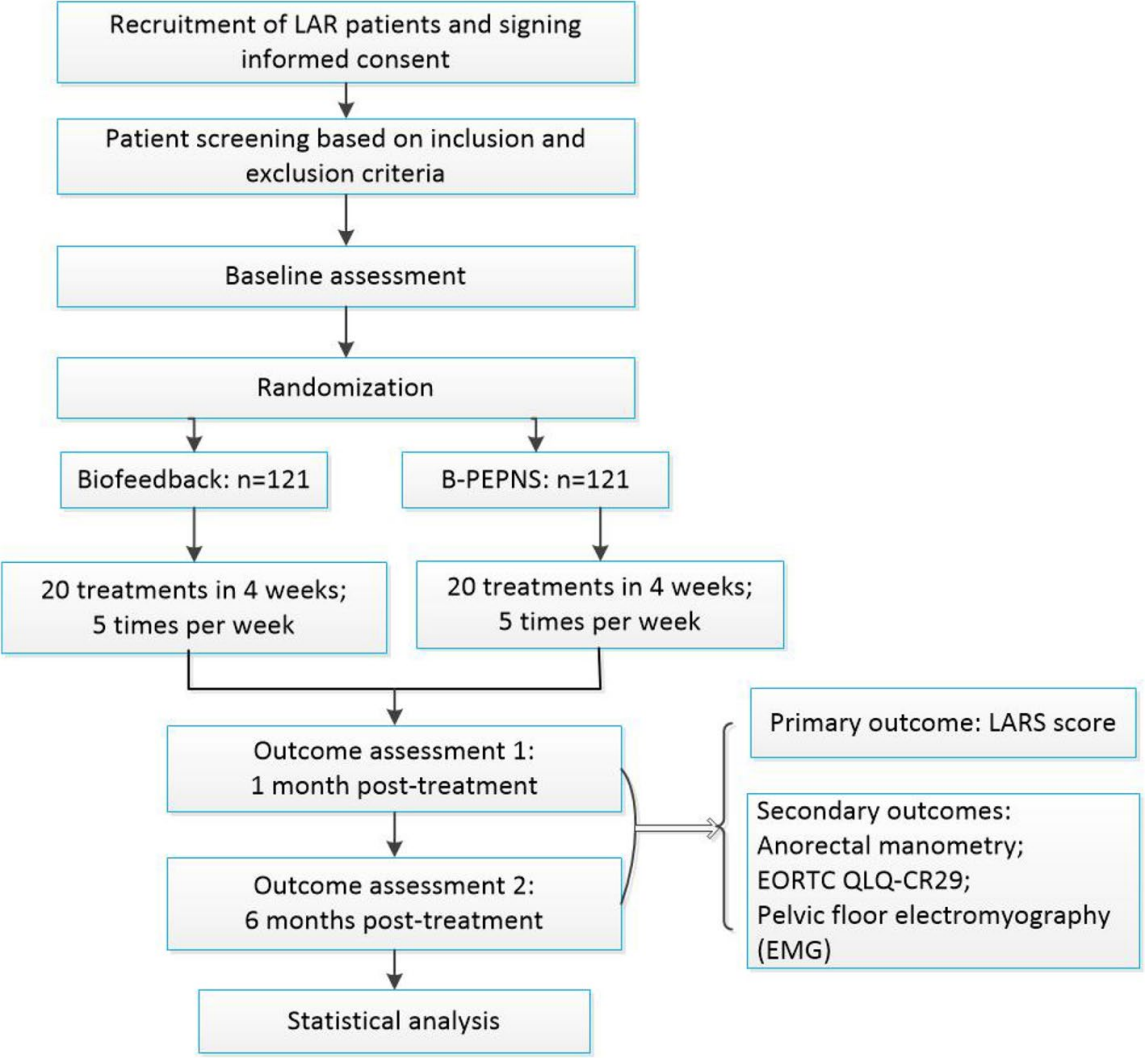
Flow chart of the study participants

This is a two-center, randomized controlled, partially blinded superiority study with a two-group parallel design, wherein data from two separate groups will be analyzed over an 8-week treatment period (from February 5, 2023, to January 31, 2025). In total, 242 LARS patients will be randomly allocated to the intervention and control groups in a 1:1 ratio. All procedures are detailed in Fig. 1 according to the Standard Protocol Items: Recommendations for Interventional Trials guidelines. [21]

## Methods: participants, interventions, and outcomes

### Study setting {9}

Patients will be recruited from two tertiary hospitals: Sir Run Run Shaw Hospital (SRRH), affiliated with the Zhejiang University School of Medicine, and the First Affiliated Hospital of Zhejiang Chinese Medical University. Biofeedback sessions will take place at SRRH, while Percutaneous Electrical Pudendal Nerve Stimulation will be administered at the First Affiliated Hospital of Zhejiang Chinese Medical University. Evaluations will be conducted at both institutions.

### Eligibility criteria {10}

#### Inclusion criteria

1. Age between 18 and 80 years of any sex
2. Presence of persistent defecation dysfunction for at least 1 month following laparoscopic-assisted anal preservation radical surgery for CRC, including cases involving temporary ileal stoma retraction surgery
3. Adherence to the LARS diagnostic criteria outlined in the international LARS expert consensus [3]
4. Normal preoperative anal function examination without any preexisting defecation dysfunction
5. Good communication skills and capability to engage actively in intervention procedures and questionnaire surveys

#### Exclusion criteria

1. Prior inflammatory bowel disease, constipation, irritable bowel syndrome, or other conditions potentially influencing bowel function
2. Permanent stoma placement for reasons unrelated to CRC surgery
3. Long-term medications that may significantly affect bowel function
4. Concurrent severe conditions affecting the heart, liver, kidneys, nervous system, or other organs
5. Recurrence or distant organ metastasis

### Who will take informed consent? {26a}

The fully trained designated researchers (XJZ, FW, DM, LW, XCP) who are present on-site will be responsible for obtaining informed consent from participants.

### Additional consent provisions for collection and use of participant data and biological specimens {26b}

On the consent form, participants are requested to sign indicating their acknowledgment of having received both written and oral information regarding the purpose, methodology, benefits, and risks associated with participating in the trial, as well as their voluntary agreement to participate. Participants are explicitly informed that they retain the right to withdraw their consent at any time without forfeiting any entitlement to treatment, present or future. Moreover, they are queried about their consent for data utilization in the event of withdrawal from the trial. This trial does not entail the collection of biological specimens for storage.

## Interventions

### Explanation for the choice of comparators {6b}

Both the control group and the intervention group will receive the same duration of biofeedback therapy, but the intervention group will also receive additional PEPNS therapy. This study will demonstrate the synergistic effects of PEPNS in alleviating LARS symptoms and improving patients’ quality of life in conjunction with biofeedback.

### Intervention description {11a}

(1) Intervention group (B-PEPNS)

#### Patients in this group will receive a combined treatment of biofeedback and PEPNS

Biofeedback Therapy: Patients will be positioned in a semi-supine stance at a 120° angle, maintaining relaxed arms and extended legs. Following lubrication, electrode probes will be carefully inserted into the anal canal and rectum, linking to a biofeedback stimulator. Patients will receive guidance to engage specific muscles—primarily the anal and pelvic floor muscles—while consciously avoiding the activation of the abdominal or thigh musculature. The therapy regimen will involve a dual-step process with initial training (Kegel template training) comprising controlled contractions for 10 s, followed by a 10-s rest period, guided by pelvic floor EMG data. Subsequent sessions will include electrical stimulation training, contingent upon preset thresholds correlated with pelvic muscle contractions. This 30-min therapy session will be conducted once daily from Monday to Friday over 4 weeks, with rest on the weekends.

PEPNS: Patients will be asked to assume a prone position. Four specific points along the caudal sacrum will be selected in alignment with the pudendal nerve direction. The puncture sites have been described in detail in the illustration in our previous study [22]. We will target the upper points (adjacent to the sacrococcygeal joint symmetrically on both sides) and lower points (located 1 cm from the coccyx tip on each side). Long acupuncture needles (0.4 mm × 100 mm; Suzhou Shenlong Medical Apparatus Factory, Suzhou, China) will be vertically inserted 75–90 mm deep at the upper points and diagonally toward the lateral side (in the direction of the ischiorectal fossa) at a depth of 90–95mm at the lower points. These needles will be connected to electrodes on a G6805 electro-acupuncture apparatus to deliver continuous electrical stimulation at a frequency of 2 Hz and intensity adjusted to each patient’s tolerance. Each PEPNS session, lasting 60 min, will be conducted Monday to Friday for 4 weeks, with rest on the weekends.

(2) Control group (biofeedback alone)

Patients in this group will exclusively undergo biofeedback treatment, following the same procedures detailed previously for the intervention group.

Licensed physical therapists with at least 3 years of experience will deliver biofeedback treatment for both the intervention and control group patients. Acupuncturists with a minimum of 2 years of acupuncture practice will perform PEPNS for the intervention group. Since biofeedback and PEPNS are part of their routine practice, they do not require additional training.

### Criteria for discontinuing or modifying allocated interventions {11b}

If a needlestick infection occurs during PEPNS, the treatment will be delayed, and antibiotics will be administered. If symptoms improve within 1 week, treatment will continue. If symptoms do not improve within 1 week, or if another infection occurs after improvement, treatment will be terminated. Meanwhile, this should be reported as an adverse event. And if any other unforeseen serious side effects occur, the intervention will also be terminated.

### Strategies to improve adherence to interventions {11c}

Adherence enhancement strategies will be implemented throughout the trial period. After 1 week of treatment, all patients will be directly scheduled for their next week’s treatment session. At the conclusion of each weekly treatment session, we will engage in discussions with patients regarding their treatment experience and remind them of the importance of adhering.

### Relevant concomitant care permitted or prohibited during the trial {11d}

Throughout the trial period, participants with background diseases, regardless of their allocation, are allowed to take relevant medications as needed. However, participants, regardless of their allocation, are prohibited from other treatments related to LARS, such as oral loperamide and enema.

### Provisions for post-trial care {30}

We will provide free medical treatment to intervention-related harms, such as needle-related infections.

### Outcome {12}

Before commencing the treatment regimen, comprehensive patient information will be compiled, including demographic data, tumor metrics, surgical specifics, and any prior radiotherapy or chemotherapy received. Patients will undergo evaluations using the LARS and European Organization for Research and Treatment of Cancer Quality of Life Questionnaire-Colorectal 29 (EORTC QLQ-CR29) scoring systems along with anorectal manometry (ARM) and pelvic floor EMG assessments. These evaluations will be conducted at the following three key times: baseline, post-intervention (at 1 month), and during follow-up (at 6 months).

#### Primary outcome

The primary outcome will be defined as the improvement rate. This will be determined by assessing the change in the LARS scores for each patient. The improvement rate will be calculated as follows:

Improvement rate (%) = [(LARS score before treatment − LARS score after treatment) ÷ total score before treatment] × 100

The treatment will be considered effective if the improvement rate surpasses 25%.

#### Secondary outcomes

1. ARM: Parameter measures, including resting pressure, maximum contraction pressure, defecation threshold, and maximum tolerance capacity of the anal canal, will be recorded.
2. EORTC QLQ-CR29 [23]: The EORTC QLQ-CR29 questionnaire is a commonly used tool for assessing the quality of life of CRC patients. It comprises 29 items categorized into six core dimensions (urinary problems, abdominal and pelvic pain, bowel problems, fecal incontinence, anxiety, and body image), four sex-related dimensions (male sexual activity, female sexual activity, impotence, and sexual intercourse difficulties), and seven standalone dimensions (bloating, dry mouth, hair loss, taste problems, skin problems, embarrassment about colostomy, and stoma care problems). Each question is rated on a specific scale, with a total score of 100. A higher score indicates more severe symptoms experienced by the patient, resulting in a greater impact on their quality of life.
3. Pelvic floor EMG: The EMG assesses various parameters, including baseline wave amplitude, rapid contraction pressure, sustained contraction pressure variability, endurance contraction pressure variability, and post-baseline wave amplitude. These parameters are measured using the biofeedback treatment device to investigate the potential functional improvements in the puborectalis and anal sphincter muscles.

### Participant timeline {13}

**Fig. 2:**
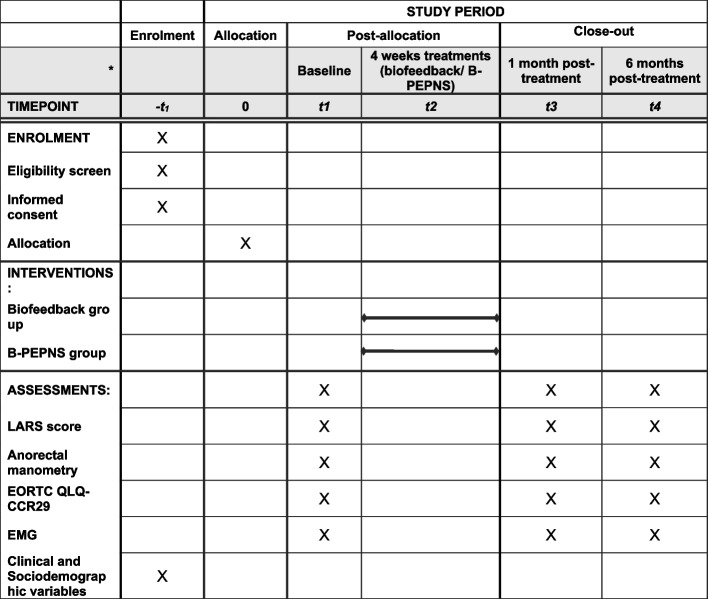
SPIRIT schedule of enrollment, intervention, and assessment

The participant timeline is outlined in the SPIRIT figure (Fig. 2).

### Sample size {14}

Based on a prior study [10] involving 45 patients in each group (efficacy rate, 65.6%:46.7%), the required sample size for this study was computed utilizing the Power Analysis and Sample Size 15 software (NCSS Statistical Software, UT, USA). A sample size of 210 patients was determined to achieve a power (1-beta) of 0.80 and an alpha (significance level) of 0.05, with group allocation in a 1:1 ratio. Factoring in potential dropouts (15% of the participants), the total sample size was adjusted to 242 in total (*n* = 121 in each group).

### Recruitment {15}

We aim to recruit 242 patients diagnosed with LARS, who meet eligibility criteria, from the inpatient and outpatient departments of the two hospitals. Our recruitment strategy includes targeted posters displayed in both hospital facilities and dissemination of health education brochures to potential participants. We will leverage mobile applications to disseminate recruitment information efficiently.

## Assignment of interventions: allocation

### Sequence generation {16a}

Using SAS software (9.3), the Clinical Evaluation and Analysis Centre at SRRH will randomly allocate 242 participants in a 1:1 ratio to the intervention group (undergoing B-PEPNS) or the control group (undergoing biofeedback alone). Professionals overseeing allocation will not be directly involved in the study.

### Concealment mechanism {16b}

Sequentially numbered opaque envelopes will conceal the random allocation, keeping it confidential from other research staff.

### Implementation {16c}

During the post-baseline assessment, an impartial staff member will open the envelope in the participants’ presence, assigning them to the intervention or control group. Simultaneously, the operators will receive information about the participant’s allocation. The recruitment staff and clinical interviewers will remain unaware of the group allotments to uphold study integrity.

## Assignment of interventions: blinding

### Who will be blinded {17a}

Throughout the trial process, data collectors, statisticians, and telephone follow-up personnel will be blinded. However, the operators will be aware of the group allocations but will not be involved in data recording, statistical analysis, or follow-up procedures. They will operate independently without any communication.

### Procedure for unblinding if needed {17b}

If a serious adverse event occurs, the participant’s allocation will be unblinded. The incident will be promptly reported to the Ethics Committee.

## Data collection and management

### Plans for assessment and collection of outcomes {18a}

Licensed therapists with at least 3 years of experience will conduct biofeedback treatment for both the intervention and control group patients. Acupuncturists conducting PEPNS for the intervention group will have a minimum of 2 years of acupuncture practice. A 2-day training session led by senior research team members will prepare therapists, acupuncturists, and assessors on all aspects of the study from diagnosis to data collection. Inspectors at each center will monitor patient enrollment, treatments, and data quality.

### Plans to promote participant retention and complete follow-up {18b}

Strategies to enhance participant retention and ensure completion of follow-up: Following each week of treatment, patients will be automatically scheduled for their next session. Additionally, patients will receive a weekly reminder via SMS on their mobile phones regarding their upcoming treatment appointment.

### Data management {19}

Data will be recorded on paper and entered into a secure electric data management system (developed by an independent data management agency, Linkermed Technology, Beijing, China) for error checks. All records will be stored safely for at least 10 years after the study while ensuring patient privacy. Personal data will be kept separate from the analysis. Both the intervention and control groups will be assigned anonymous numerical codes (1, 2) to blind the individual conducting the statistical analysis. The collected data will only be used for this research.

### Confidentiality {27}

Personal information will be retained solely by the therapist within a secure electronic data management system. All records will be securely stored for at least 10 years after the study to safeguard patient privacy.

### Plans for collection, laboratory evaluation, and storage of biological specimens for genetic or molecular analysis in this trial/future use {33}

Not applicable, no samples were collected.

## Statistical analysis

### Statistical analysis plan (SAP) for primary and secondary outcomes {20a}

#### Overview

The primary and secondary outcome analyses will be conducted using IBM SPSS Statistics version 27.0. A comprehensive SAP has been established to ensure robust and accurate interpretation of the data. This plan adheres to the guidelines published in JAMA [24] and incorporates principles for the prospective reporting of statistical analysis plans for RCTs [25].

#### Data processing and preliminary steps

1. Normality test: Data will be assessed for normality using the Shapiro-Wilk test.
2. Handling baseline imbalances: If baseline characteristics and outcome measures show imbalances, analysis of covariance (ANCOVA) will be applied to adjust for these differences.
3. Data presentation: Normally distributed continuous variables will be presented as means ± standard deviations (SD). Non-normally distributed or ordinal variables will be presented as medians with interquartile ranges (IQR). Categorical variables will be presented as counts and proportions.
4. Missing data: The last observation carried forward (LOCF) method will be used to handle missing data points. Other rules are referenced in Item 20c.

#### Primary outcome analysis

The primary outcome of this study is the change in LARS scores.

1. Descriptive statistics: continuous variables (normally distributed): means ± SD. Continuous variables (non-normally distributed): medians with IQR. Categorical variables: Counts and proportions.
2. Inferential statistics: Student’s *t*-test: used for analyzing normally distributed primary outcome measures (LARS scores). Paired* t*-tests: to compare pre-treatment and post-treatment improvement rates within each patient for LARS scores. Independent sample *t*-tests: to compare improvement rates in LARS scores between the intervention and control groups. Mann-Whitney *U* test: For inter-group comparisons of non-normally distributed LARS score data. Wilcoxon signed-rank test: to compare pre-treatment and post-treatment improvement rates in LARS scores for non-normally distributed data within each patient. Intergroup rank sum test: To assess inter-group differences in ordinal data.

#### Secondary outcome analysis

1. ARM and EMG parameters, EORTC QLQ-CR29 scores: Analyzed using the same statistical methods as the primary outcomes.
2. Statistical tests: continuous data: analyzed using Student’s *t*-tests, Mann-Whitney *U* tests, paired *t*-tests, and Wilcoxon signed-rank tests as appropriate. Categorical data: analyzed using chi-square tests or Fisher’s exact tests as appropriate.
3. Reporting and significance: all *p*-values reported will be two-tailed. Confidence intervals will be set at the 95% level. Statistical significance will be defined as *p*-values < 0.05.

#### Additional notes

Detailed results and any supplementary analyses will be made available upon request.

By adhering to this structured SAP, we aim to provide clear, reliable, and interpretable results for both the primary and secondary outcomes of this study.

### Interim analyses {21b}

This trial is not subject to interim analyses.

### Methods for additional analyses (e.g., subgroup analyses) {20b}

Subgroup analysis will be performed for the radiotherapy group. Participants who have undergone pelvic radiotherapy related to rectal cancer will be included in the radiotherapy group.

### Methods in analysis to handle protocol non-adherence and any statistical methods to handle missing data {20c}

In cases where the lost-to-follow-up rates are below 20%, we will conduct an intention-to-treat analysis. Missing values will be replaced using single imputation procedures with the group mean.

### Plans to give access to the full protocol, participant-level data, and statistical code {31c}

The datasets analyzed during this trial can be made available from the corresponding author upon reasonable request.

## Oversight and monitoring

### Composition of the coordinating center and trial steering committee {5d}

GYC, XJZ, and SC, the lead study coordinators, comprise the trial steering committee. Additionally, three independent members from the Clinical Trials Office of Sir Run Run Shaw Hospital, along with the three lead coordinators, will serve as the coordinating center for this study. These committees will convene monthly to assess the study’s progress and address any financial or technical issues that may arise.

### Composition of the data monitoring committee, its role, and reporting structure {21a}

The clinical trial will not incorporate an external data monitoring committee due to the utilization of therapies commonly practiced by pelvic physical therapists and acupuncturist and assessments frequently administered to rectal cancer patients. However, an internal committee will be established to monitor preliminary safety data. This committee will comprise the researchers responsible for assessing the primary outcomes and the principal investigator. They will conduct ongoing preliminary data analyses to assess safety and efficacy. In the event of any reported adverse events, this committee will seek analysis and recommendations from an external surgeon with expertise in treating rectal cancer. The data monitoring group operates independently from the funder and sponsor.

### Adverse event reporting and harms {22}

The study will monitor for PEPNS-related adverse events (AEs), such as needle-related infections, and report them to the Ethics Committee. We will document the site, extent, treatment process, and recovery time of each AE, and assess the causality between AEs and the intervention. AEs will be used as safety outcome measures to evaluate the safety of the intervention.

### Frequency and plans for auditing trial conduct {23}

Auditing will be conducted only in case the funder or Ethical Committee requires.

### Plans for communicating important protocol amendments to relevant parties (e.g., trial participants and ethical committees) {25}

In this trial, important protocol modifications must be approved by the trial registry and the Ethical Committee.

### Dissemination plans {31a}

The study findings will be submitted for publication in peer-reviewed journals, irrespective of whether they support or challenge the initial study hypothesis.

## Discussion

The management of LARS is crucial considering its high prevalence and severe impairment of the quality of life [26]. The treatment timing is important, and early intervention for LARS is preferred as it is better for neural functions [27]. Therefore, as per our protocol, our intervention can start 1 month postoperatively for anal preserve rectal cancer or temporary ileal stoma retraction.

Opting for a combined approach of biofeedback and PEPNS for treating LARS is grounded in their potential synergistic action in LARS management as well as their high operational feasibility. Biofeedback, utilizing superficial electrodes and anal internal sensing probes, accurately detects pelvic muscle movements, providing effective guidance to patients during pelvic floor exercises. It also promotes pelvic muscle rehabilitation through superficial electrical stimulation. On the other hand, PEPNS precisely locates the perineal nerve positions via percutaneous needling, directly stimulating these nerves to increase the resting sphincter pressure, thereby enhancing bowel control. Biofeedback is a routine outpatient procedure conducted in many tertiary hospitals, whereas PEPNS implementation only requires an acupuncture apparatus and basic training for physicians in needling techniques, which can be easily sought in Chinese medicine hospitals. Hence, the execution of this study is comparatively straightforward.

The symptoms of LARS tend to be more severe in the initial months after surgery, especially in the first 4 months, followed by a gradual improvement and stabilization between 6 months to 1 year [4, 5]. Therefore, our assessments not only include a short-term evaluation 1 month after treatment but also a long-term evaluation after 6 months. This is aimed at minimizing potential biases due to natural symptom variations over time. Additionally, based on the results from the 6-month long-term evaluation, we will determine the necessity for further assessment after 1 year.

The selected outcome measures are based on their validity and reliability. The LARS score has been tested in the Chinese population and measured bowel dysfunction in patients after CRC surgery [23, 28]. This scoring system measures various aspects of postoperative bowel function, such as fecal incontinence, urgency of bowel movements, and bowel frequency, aiding physicians in evaluating the recovery and quality of life of patients postoperatively. A higher score indicates a more severe impact on the patient’s postoperative bowel function. ARM, the anorectal pressure measurement device, evaluates the strength, coordination, and reflex mechanisms of the anal sphincter muscles, pivotal for maintaining continence. [29]

The EORTC QLQ-CR29 is an evaluative tool used to gauge the multifaceted dimensions concerning treatment aspects and their influence on the daily lives of CRC patients. This comprehensive questionnaire covers distinct areas, encompassing symptomatic experiences, functional constraints, and emotional well-being directly associated with the ailment and its therapeutic interventions. Through its systematic inquiry method, the EORTC QLQ-CR29 assists in the meticulous assessment and resolution of the diverse array of challenges encountered by individuals undergoing CRC treatment. Its use helps in significantly fostering a more comprehensive and integrated approach toward patient care and therapeutic management strategies. [30]

Pelvic floor EMG is a diagnostic method assessing pelvic muscle activity via electrical signals. It evaluates the muscle strength, coordination, and relaxation patterns in pelvic floor disorders, such as incontinence. EMG aids in facilitating tailored treatments by providing precise muscle function insights, contributing to improved patient care.[31] 

Nevertheless, it is crucial to acknowledge certain limitations of this study, including potential variability in participant adherence and the absence of long-term follow-up data. Additionally, the inability to blind participants and therapists to the chosen treatment methods might introduce bias. However, efforts will be made to ensure independent data collection and processing, aiming to mitigate potential biases as much as possible.

In conclusion, this study protocol introduces a promising avenue for treating LARS, thus addressing a critical gap in the therapeutic options. The combination of biofeedback and PEPNS offers a potential breakthrough in non-surgical interventions. The findings of this RCT could provide clinicians with a more effective and patient-friendly approach when managing LARS.

## Trial status

This is protocol version 1, 1 January 2022. Trial recruitment commenced on February 5, 2023, and is scheduled to conclude by January 31, 2025.

## Data Availability

The datasets generated and/ or analyzed during the study will be available on reasonable request. Data can be obtained by contacting the main researcher. Email for requests: breezehilly@hotmail.com.

## Abbreviations

LARS: Low anterior resection syndrome
PEPNS: Percutaneous electrical pudendal nerve stimulation
B-PEPNS: Biofeedback with percutaneous electrical pudendal nerve stimulation
ARM: Anorectal manometry
EMG: Surface electromyography
EORTC QLQ-CR29: European Organization for the Research and Treatment of Cancer Quality of Life Questionnaire-Colorectal 29
